# Using Deep Learning and B-Splines to Model Blood Vessel Lumen from 3D Images

**DOI:** 10.3390/s24030846

**Published:** 2024-01-28

**Authors:** Andrzej Materka, Jakub Jurek

**Affiliations:** Institute of Electronics, Lodz University of Technology, 90-924 Lodz, Poland; jakub.jurek@p.lodz.pl

**Keywords:** blood vessels, lumen quantification, centerline, deep learning, 3D images, B-splines, NURBS, tubular objects

## Abstract

Accurate geometric modeling of blood vessel lumen from 3D images is crucial for vessel quantification as part of the diagnosis, treatment, and monitoring of vascular diseases. Our method, unlike other approaches which assume a circular or elliptical vessel cross-section, employs parametric B-splines combined with image formation system equations to accurately localize the highly curved lumen boundaries. This approach avoids the need for image segmentation, which may reduce the localization accuracy due to spatial discretization. We demonstrate that the model parameters can be reliably identified by a feedforward neural network which, driven by the cross-section images, predicts the parameter values many times faster than a reference least-squares (LS) model fitting algorithm. We present and discuss two example applications, modeling the lower extremities of artery–vein complexes visualized in steady-state contrast-enhanced magnetic resonance images (MRI) and the coronary arteries pictured in computed tomography angiograms (CTA). Beyond applications in medical diagnosis, blood-flow simulation and vessel-phantom design, the method can serve as a tool for automated annotation of image datasets to train machine-learning algorithms.

## 1. Introduction

Vascular diseases, characterized by either blocked or excessive blood supply to organs and tissues, are among the most serious health challenges globally [1], leading to life-threatening conditions including stroke or heart attack. They are associated with abnormalities in blood vessel lumen geometry, such as stenoses caused by pathological deposition of atherosterotic plaque inside the vessel [2]. The diagnosis and treatment of vascular diseases requires accurate lumen geometry quantification, in a non-invasive way where possible [3]. Three-dimensional imaging is the main technique used to acquire quantitative information about the vasculature [4]. Example modalities include magnetic resonance angiography (MRA) which can be either blood-flow-dependent (time-of-flight (TOF) or phase contrast angiography (PCA)) or flow-independent [5,6]. An invasive alternative is computed tomography angiography (CTA) [7]. Vasculature images can be nonenhanced or contrast-enhanced (CE), which involves injecting a contrastive substance into the blood circulatory system [8]. Vascular ultrasonography (US) is another imaging technique with good potential [9]. However, modeling the US image formation requires different theoretical treatment [10] to that applicable to MR and CT volumes. Further discussion of US imaging lies beyond the scope of this paper.

Parametric evaluation of the shape of the blood vessel lumen requires identification and quantification of 3D image regions representing the relevant part of the circulatory system. This is not a trivial task, for several reasons. Firstly, the vasculature exhibits a complex tree-like structure of highly curved branches of different diameters [11,12] (p. 9). These diameters range broadly, from 30 mm in the aorta and vena cava to as small as 30 µm in venules and arterioles [13]. At the same time, the spatial resolution of imaging systems is limited. For instance, in clinical scanners, the spatial resolution is limited to cuboids (voxels) with a minimum sidelength of 0.2 mm. Thus, the thin regions of vessels are heavily blurred in the image, and the thinnest of them are not visible. To enhance the resolution, various technical solutions are employed to reduce the voxel volume [14,15]. However, this causes a decrease in the signal collected from each voxel, and as a consequence increases the impact of random noise as a component of the image intensity. The presence of random noise increases the uncertainty of assessing intensity levels inside the lumen, the vessel wall, and the surrounding background, adversely affecting the lumen geometry measurements (Appendix A). Image artifacts further complicate vessel image analysis.

**Figure 1:**
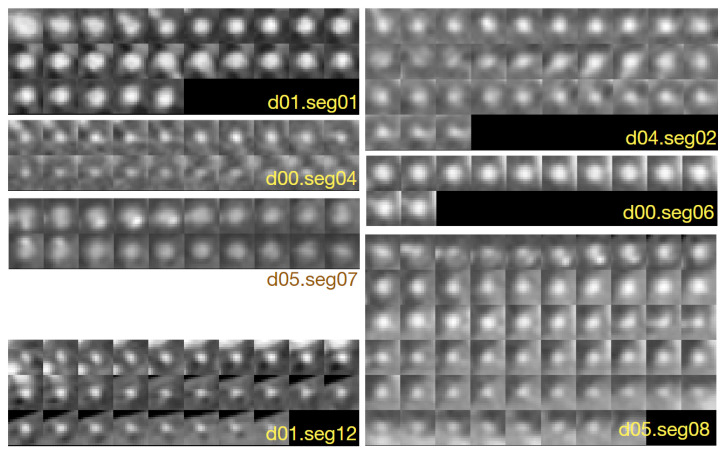
Example 15 × 15-pixel cross-sections of coronary artery segments in CAT08, pixel size 0.45 × 0.45 mm.

Typically, the vessels of interest are closely surrounded by other arteries, veins and tissues. Thus, the background region features uneven image intensity and the spatially blurred signals from different regions overlap (Figure 1). Moreover, the intensity inside the vessel walls is not constant. The plaque depositions, especially those that are calcified, exhibit different physical properties to the blood or to the contrastive medium [16]. Under these conditions, classical, numerically efficient segmentation through thresholding becomes highly inaccurate. These factors make the task of vessel quantification in 3D images especially challenging for radiologists. Manual vessel delineation in 3D images is tedious, time-consuming, and error-prone [17]. Its adequacy depends much on the reader’s experience and their level of fatigue. There is a strong need to develop automated techniques for accurate, fast, and objective vascularity evaluation from volumetric image data [18].

Numerous methods of lumen segmentation and quantification have been proposed over the last few decades [17,18,19]. Research in this area has further intensified with the development of deep learning as an approach to image segmentation [20,21]. There are two main approaches to vascular structure quantification in 3D images [22,23]:

- Three-dimensional segmentation-based quantification;
- Model-fitting-based 2D lumen cross-sections quantification along an approximate centerline.

The result of direct 3D image segmentation is another image. In its basic form, this image is a grid with the same spatial resolution as the original volume, where the nodes (e.g., the centers of the voxels) are attributed binary intensity values. Some of these voxels are marked by a label indicating that they represent the lumen. All the others are labeled as background. The boundary of the lumen region, which is smooth and continuous in the physical space, is discretized during image acquisition and so it remains after segmentation. For random boundary location in space, the variance of the discretization error can only be reduced by increasing imaging resolution (Appendix A) usually at the price of increased noise.

Thus, when binary segmented, the lumen surface exhibits a stair-like or voxelized form. It requires additional spatial smoothing for visualization or to build a geometric model for blood flow simulation. However, part of the information about the actual course of this continuous surface, which is needed for faithful reconstruction, is lost in the binary segmentation step, when the continuous intensities are replaced by two-level values. From this point of view, image segmentation, although intuitive, is an unnecessary processing step. Moreover, some segmentation algorithms involve time-consuming iterative calculations [24,25].

Here, we focus on the second approach to estimating lumen geometry parameters, from 3D image cross-sections. These cross-sections are computed as 2D images on planes perpendicular to the vessel centerline, which is approximated by a smooth curve in 3D space [26]. Various algorithms for the centerline extraction are available [27,28]. Normal vectors to the centerline define the cross-section planes. The cross-section images are obtained through 3D discrete image interpolation and resampling. The center of the cross-section image grid is usually set to lie on the centerline. The contours of the lumen are delineated, either semi-manually [29] or automatically [26]. For automated contour delineation, a 2D image formation model is defined. This model accounts for image smoothing by an equivalent scanner impulse response, either one-dimensional along radial lines [26,30] or two-dimensional over the image plane [31], as well as for the random noise. The effect of smoothing, naturally featured by imaging sensors, plays an important role in the chosen approach. Namely, smoothing converts distances between image points and the lumen edge to image intensity variations. Thus, more relevant information is available (in the transition region around the edge) compared to voxelwise thresholding (Appendix A). The model is fitted with the use of the least-squares (LS) algorithm. This involves long-lasting iterative minimization of a nonlinear error function and is likely to become stuck in its local minima, depending on the initial parameter values.

**Figure 2:**
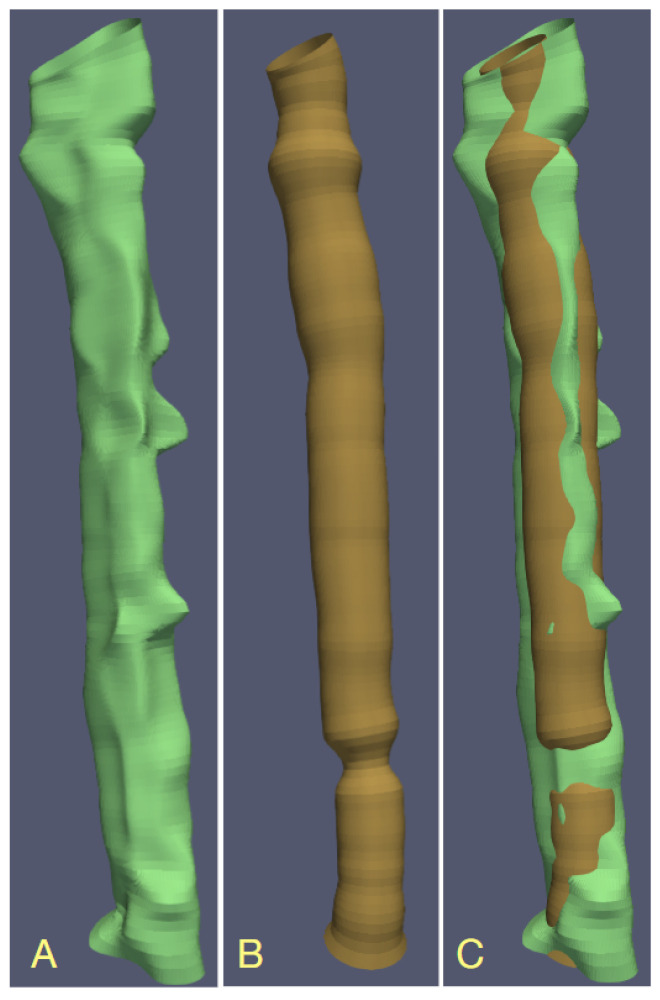
Visualization of the PAVES b14 branch of vein–artery lumen based on contours obtained with different LS-identified image formation models, (**A**) B-spline contours, (**B**) circular lumen boundaries, (**C**) overlay of the surfaces in (**A**,**B**).

We use LS fitting as a reference method and apply the convolutional neural network (CNN) driven by 2D cross-section images for fast estimation of the model parameters [31,32]. The contours found at predefined increments along the centerline arc are lofted to build the geometric models, e.g., for visualization (Figure 2). Lofting is a technique used in computer-assisted design programs to create 3D surfaces or solid objects [29]. They are formed from sequences of cross-section curves—the contours of the lumen, in our case.

Past works that followed the modeling approach incorporate circular or elliptical cylinders as abstract geometric objects that represent the vessel lumen in the image space. In fact, the actual shape of the lumen contours significantly deviates from these idealized figures (Figure 2). This happens in particular in the vessels narrowed by atherosclerotic plaque [33]. The novelty of our method lies in using B-splines—parameterized curves that accurately represent the natural lumen shapes in normal and pathological vasculature—as part of the image formation model. Although B-splines have been used previously for blood vessel contouring [34,35], they were fitted to the approximate, spatially discretized surface of the lumen region in binary segmented 3D image. In our approach, the fitting process takes place in the image at full bit depth intensity instead of in the discretized spatial domain. Innovatively, the lumen contour parameters are estimated by a neural network, providing increased speed and robustness compared to LS fitting. The use of B-splines makes our method compatible with the isogeometric approach [36] to image analysis, test object (physical phantoms) design and computational blood flow simulation.

## 2. Materials and Methods

To illustrate the properties and capabilities of the proposed lumen modeling method, the following real-life and synthetic images were used:

- Lower-extremities MRA volumes available within the PAVES Grand Challenge [37];
- Contrast-enhanced CTA of coronary arteries collected and annotated by a team of researchers at the Erasmus Medical Center in Rotterdam [38], provided within the framework of the MICCAI 2008 Coronary Artery Tracking Challenge (CAT08) [28];
- Computer-synthesized cross-section images generated for purposes of this research.

These datasets are characterized in the following subsections.

### 2.1. PAVES MR Dataset

Steady-state MR contrast-enhanced volumes, available as part of the PAVES dataset, are high spatial resolution images which, in principle, allow evaluation of the shape of the artery lumen, e.g., for the purpose of identifying stenosis. However, these images take several minutes to acquire. During this rather long time, both the arteries and veins become filled with the gadolinium-based contrast medium. As a result, the intensities of these regions show similar values, making the arterial regions difficult to identify and distinguish from the neighboring veins. The arteries are smaller in diameter, and their cross-sections are close to circular, except for pathological stenosed vessels featuring wall-thickening. On the other hand, veins typically have oval shapes and are located in pairs next to an artery (Figure S1). Thus, the cross-sections of artery–vein complexes do not exhibit circular shapes. They are non-circular, although their boundaries are smooth. As highlighted in [39], veins can be used by surgeons to “bypass” a blocked artery, provided they are of the correct diameter and length. Therefore, delineating veins and arteries in high-resolution GdCE (gadolinium contrast-enhanced) MR images is a valid medical image processing task, formulated as a Grand Challenge [37].

The research problem defined in this example is to evaluate the suitability of the proposed method to delineate boundaries of the veins and arteries, given their steady-state GdCE MR volume. The PAVES dataset 5 was selected for this purpose, as the clinical significance of its properties was demonstrated in [39]. To obtain the cross-sections, the centerlines of the blood vessel regions were extracted and smoothly approximated by application of the following 3D image preprocessing steps [26]:

- Multiscale “vesselness” filtering [27,40] to enhance the blood vessels regions [41];
- Vesselness map thresholding to obtain binary lumen representation;
- Binary region thinning to produce a skeleton;
- Skeleton parsing [42] to identify the blood vessel tree branches between bifurcations;
- Approximating the skeleton branches by a differentiable function in 3D (to initialize their centerline).

**Figure 3:**
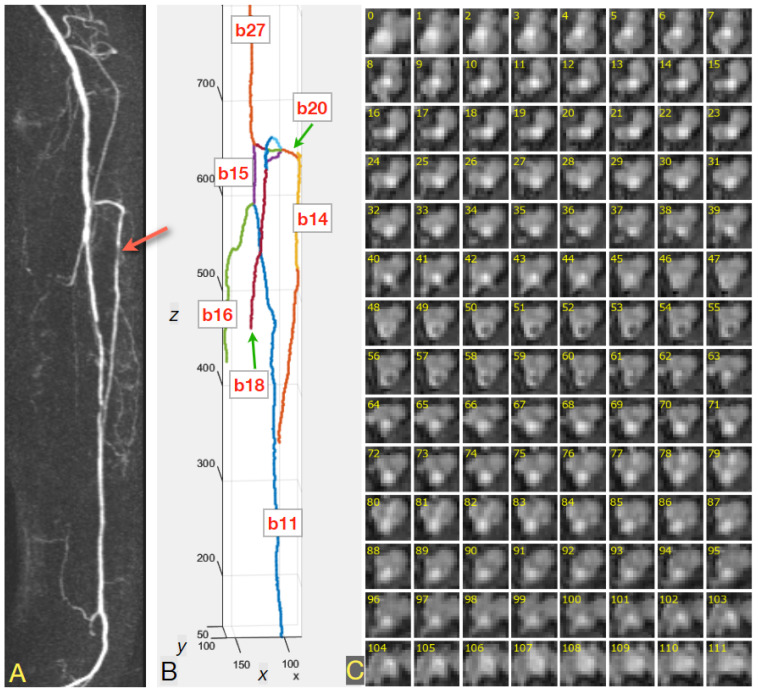
(**A**) Maximum intensity projection for PAVES dataset 5, showing TWIST (subtracted time-resolved acquisition) volume on the axial plane, left volunteer extremity. The arrow indicates a stenosis in the anterior tibial artery. (**B**) Binary skeleton of the blood vessels, after parsing. The tibial artery branch was assigned code b14. (**C**) Mosaic of 112 numbered cross-sections of the tibial artery, taken at 0.5-mm intervals along the centerline. An example of a coronal slice MRI of the volunteer’s right leg is shown in Figure S1.

Figure 3A shows a maximum intensity projection on the axial plane of the PAVES dataset 5 TWIST (subtracted time-resolved acquisition) volume, for left volunteer extremity. The arrow indicates a stenosis in the anterior tibial artery. The above preprocessing algorithm was applied for the TWIST 3D image. The parsed skeleton is illustrated in Figure 3B. The tibial artery segment b14 was selected for further analysis, among other vessel-tree branches enumerated by the skeleton parsing algorithm.

Tangent vectors to the smooth vessel centerline curve were computed at a number of consecutive points at distances of about 0.5 mm from each other. Together with the binormal vectors, these tangent vectors define the Frenet-Serret frame at each point. This frame often becomes rotated around the tangent vector, causing an undesirable twist in the surface of an elongated object. A rotation minimizing frame algorithm [43] was then used to compute the vessel local coordinate systems 0*xy*, in which the 2D lumen cross-sections were calculated by resampling the MR volume. These image preprocessing and analysis algorithms were implemented in both Matlab (version R2022B) and Python (version 3.10.13). The codes are available on request.

Examples of the cross-sections are presented in Figure 3C, in the form of a mosaic of 15 × 15-pixel images, where the pixel size is 1.0 mm × 1.0 mm. The consecutive lumen cross-sections show minimal twist. Basically, a cross-section contains a dark background where there is no tissue filled with blood, a medium-intensity vein region, and a bright artery blob with the highest concentration of the contrastive medium. As shown in the upper five rows of Figure 3C, the healthy arteries feature circle-like cross-sections. However, the atherosclerotic occlusions cause narrowing of the vessel lumen (row six, right half) and reduce the intensity of the artery region. As an effect of collateral blood circulation, the width of the artery lumen may be restored down the vessel, as can be seen in rows nine to fifteen (Figure 3C).

### 2.2. CAT08 Coronary CTA Dataset

We applied the proposed lumen modeling method to 3D images of a training set made available by the Rotterdam Coronary Algorithm Evaluation Framework [38,44,45]. It was designed for the development of algorithms for lumen segmentation, as well as detection and quantification of coronary artery stenoses, and provided in the MICCAI 2008 Coronary Artery Tracking Challenge (CAT08). There are 48 datasets available in the Rotterdam Challenge data repository. In 18 of the datasets, anatomical segments of the arteries (specified in [44]) have been manually annotated by three experts. These 18 sets constitute the CAT08 training set. For 78 of the training set segments, expertly annotated contours of the arteries cross-sections are available. We used this information, together with the corresponding 3D images, to train the CNN estimator and as a reference for testing. It is worth noting the three observers needed approximately 300 h to complete their annotation of the data [38].

The CT images of the Rotterdam dataset were acquired with different scanners over a period of one year, for a number of patients under observation for cardiac diseases. The average voxel size for the whole dataset is 0.32 mm × 0.32 mm × 0.4 mm. We clipped the CT volume intensity to [−300,800] HU (Hounsfield units) and normalized it to [0,1]. The volumes were resampled on the planes of marked contours, to obtain lumen cross-sections centered at the centerline points.

Figure 1 shows example cross-sections of a few segments in the CAT08 dataset, computed for a sampling interval of 0.45 mm. Segments seg01 and seg02 are consecutive anatomical parts of the right coronary artery (RCA). However, the sections of these segments presented in Figure 1 belong to different datasets and differ in appearance quite significantly. The contrast for the 25 images of seg01 is much higher and the area of its lumen region does not change as much as the lumen region for the 33 sections of seg02.

Thresholding would not be useful for lumen region segmentation of seg02. The lumen diameter noticeably decreases along the 20 sections of seg04 (from left to right, row by row) and approaches subpixel values in the bottom row. At the same time, bright regions in the background dominate the image content. The contrast of images in seg06 is good. The lumen region is well defined and its shape is close to circular. However, it shows a significant blur, similar to all other images. This property, originating in the limited spatial resolution of the CT scanners, leads to high uncertain localization of lumen boundaries, especially in noisy images.

**Figure 4:**
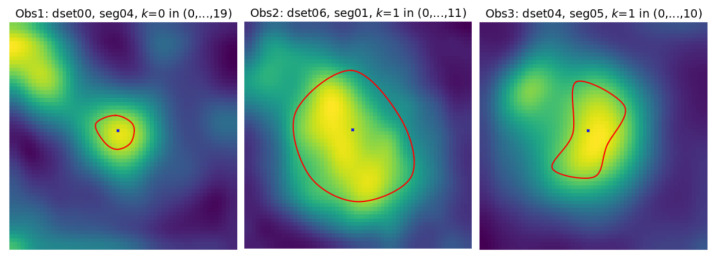
Example contours marked by the three observers on coronary artery sections in the CAT08 dataset. Cross-sections were interpolated to 60×60 pixel resolution to make their appearance similar to the example shown in Figure 4 of [38]. The pseudocolor palette was used to enhance the visibility of the intensity variations.

Segments seg07 and seg08 form consecutive parts of the left anterior descending artery (LAD). The first section (the leftmost in the upper row) of seg07 is closest to the heart, the last section (rightmost in the lower row) is the immediate neighbor of the first section in seg08. The sections in the upmost row of seg08 exhibit a reduced lumen area and bright image spots in the sixth and seventh sections, apparently caused by atherosclerotic plaque. If they become part of the lumen model as a result of image analysis, postprocessing will be needed to detect and quantify them. seg12 is the first obtuse marginal artery (OM1). Its lumen is well distinguished from the immediate background. However, a significant area is occupied by black triangular-shaped regions where the normalized intensity is close to zero (−300 HU or less in the scanner-acquired CT volumes). The typical cross-section shape of the vessel lumen marked by the observers is neither circular nor elliptical (Figure 4).

All the image nonidealities pointed out in the previous paragraph make lumen modeling a difficult task. In our numerous experiments, the LS model fitting method failed in the case of too many CAT08 arterial branches, as background regions were of locally higher intensity than the lumen. On the other hand, the proposed parameterized B-spline lumen contour model can be robustly identified by a feedforward CNN, which will be demonstrated in the Results section.

### 2.3. Image Formation Model and the Synthetic Dataset Generation

We assumed the blood vessel cross-section image centered at any point (ξ,η,ζ) in the 3D space is a convolution of the function f(x,y), which represents the lumen and its background, with h(x,y)− the imaging system effective impulse response (point spread function PSF):

(1) $$F\left(x,y\right)=\int_{-\infty }^{\infty }\int_{-\infty }^{\infty }f\left(u,v\right)h\left(x-u,y-v\right)dvdu$$

where (x,y) are image coordinates on the cross-section plane. The function h(x,y) in (1) combines the effects of the 3D image scanner impulse response and interpolation necessary for computing the cross-section intensity from the 3D image via resampling. This assumption, though idealized, is relevant to most practical situations [46].

We further assume the function f(x,y) is constant within the lumen region, surrounded by a background of different but also constant intensity. These assumptions are often not met, as mentioned before with regard to the coronary artery CE CTA images. We will demonstrate in the Results section that CNN-based lumen geometry restoration is robust to image intensity variations in the regions of interest.

We considered equidistant sampling on a Cartesian grid of points (although other sampling strategies are possible to tune the estimator properties):

(2) $$\left(x_{i}^{s},y_{j}^{s}\right)=\left(i\Delta_{s},j\Delta_{s}\right),\;i,j\in \left\{-N,\cdots ,0,\cdots ,N\right\}$$

where $\Delta_{s}$ denotes the sampling interval. The cross-section image size is (2N+1)×(2N+1). The image intensity at (i,j) is the sampled convolution (1) multiplied by lumen intensity step *b* and added to the background intensity *a*:

(3) $$I\left(i,j\right)=a+bF\left(i\Delta_{s},j\Delta_{s}\right).$$

The lumen section boundary at each centerline point $\left(\xi_{k},\eta_{k},\zeta_{k}\right)$, k∈{0,⋯,K−1} is described in this paper by a closed (periodic) B-spline curve which encloses the higher-intensity lumen region $\Omega_{k}$ in $R^{2}$:

(4) $$f_{k}\left(x,y\right)=\left\{\begin{array}{ccc} 1 & for & \left(x,y\right)\in \Omega_{k}\\ 0 & for & \left(x,y\right)\notin \Omega_{k} \end{array}\right.$$

The in-plane blur in our model is assumed to be an isotropic Gaussian [46,47,48], where *w* is a parameter:

(5) $$h\left(x-u,y-v\right)=\frac{1}{2\pi w^{2}}\exp\left(-\frac{{\left(x-u\right)}^{2}+{\left(y-v\right)}^{2}}{2w^{2}}\right).$$

The lumen contour is approximated by a parameterized curve lying on the plane of the vessel cross-section. For this purpose, we selected a B-spline curve [49,50], which is a smooth, piecewise polynomial allowing stable, locally controlled approximation of real-world data. To define a curve of this kind, it is necessary to specify a sequence of M+2, M>0, real numbers, the knots

(6) $$\left(t_{1},t_{2},\cdots ,t_{M+n}\right),\;t_{i}\leq t_{i+1}$$

which define the curve segment boundaries, M>0, and *n* as the B-spline degree. At the boundaries (internal knots), the segments are joined together to satisfy continuity constraints. We use third-degree polynomials, meaning that the first and second derivatives are continuous along the curve arclength.

The closed B-spline of degree *n* is controlled by M+n+1 points $C_{i}$, such that the first control point $C_{0}$ overlaps with the last one in the sequence. For any *t* within the B-spline segments, the curve points can be computed as [51]

(7) $$B\left(t\right)=\sum_{i=0}^{M+n}C_{i}B_{i,n}\left(t\right)\;t_{0}\leq t\leq t_{M+1}$$

The B-spline (7) is a linear combination of degree *n* basis B-splines $B_{i,n}$. These can be defined recursively using the de Boor algorithm [49]

(8) $$B_{i,0}\left(t\right)=\left\{\begin{array}{ccc} 1 & if & t_{i}\leq t_{i+1}\\ 0 & otherwise \end{array}\right.$$

(9) $$B_{i,j+1}\left(t\right)=\frac{t-t_{i}}{t_{i+j}-t_{i}}B_{i,j}\left(t\right)+\frac{t_{i+j+1}-t}{t_{i+j+1}-t_{i+1}}B_{i+1,j}\left(t\right)$$

To compute the cross-section model intensity (3), one needs to specify the boundary of the lumen region Ω in (4). This is described by a B-spline curve controlled by a sequence of points that have to be defined. In our approach, the control points for a cross-section centered at the vessel centerline are located on the radial lines extruding from the image center at *D* angles:

(10) $$\phi_{i}=\frac{2\pi i}{D},\;i\in \left\{0,\cdots ,D-1\right\}.$$

The distance in physical space from the image center to the *i*th control point $C_{i}$ is $Rd_{i}\Delta_{s}$, i∈{0,⋯,D−1}. *R* is a scale factor (fixed for the image of given size) and $d=(d_{0},d_{1},\cdots ,d_{D-1})$ is a vector of adjustable B-spline parameters. The coordinates of the control points are computed as

(11) $$C_{i}=Rd_{i}\Delta_{s}\left(\cos\left(\phi_{i}\right),\sin\left(\phi_{i}\right)\right)\;i\in \left\{0,\cdots ,D-1\right\}.$$

**Figure 5:**
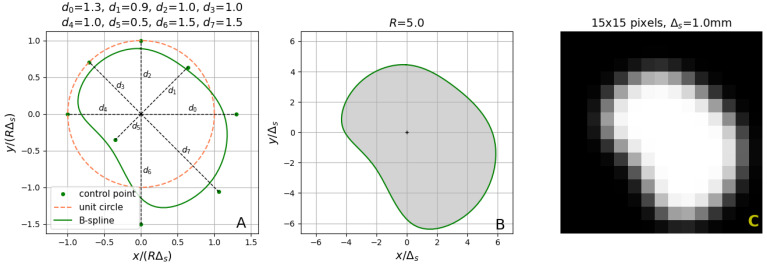
(**A**) Geometry of an eight-parameter B-spline curve normalized to the scale factor *R*. (**B**) Ideal lumen region in the cross-section image space. The shaded area represents the constant-intensity lumen region Ω. (**C**) Low-resolution noiseless image of the lumen on its background.

Based on our experience with blood vessel images of various modalities, the *w* parameter of the PSF in (5) does not change significantly within an image. Therefore, a constant value of *w* is assumed and measured separately, e.g., via analysis of appropriate edge blur. Then, there are altogether P=D+2 adjustable parameters p=(a,b,d). Combining expressions from (1)–(11), one obtains

(12) $$I\left(i,j;p\right)=a+bF\left(i\Delta_{s},j\Delta_{s},d\right).$$

where F(x,y) given by (1) is multiplied by the parameter *b* (the intensity pulse corresponding to the lumen region) and added to the background intensity *a*. Geometrical interpretation of the elements of vector d is provided in Figure 5A, for D=8. The lumen is represented in (4) by a set of points of unit intensity (the shaded area in Figure 5B), surrounded by the background points with intensity equal to zero (the white area in Figure 5B). Figure 5C shows a low-resolution image obtained as a result of sampling a blurred version of the ideal image depicted in Figure 5B.

Considering the complex shape of the region Ω, no closed-form expression for the integral (1) appears to be available. To compute 2D images for the training and testing datasets, we evaluated the right-hand side in (1) numerically, with a Python implementation of the OpenCV (version 4.6.0) library. A synthetic dataset comprising 10,000 images was created. The synthetic dataset was considered in two versions: noiseless and noisy. The noise addition procedure is described in the Results subsection devoted to the PAVES dataset.

### 2.4. Least-Squares Model Fitting for the PAVES Dataset

The aim is to estimate the parameter vector $\hat{p}=\left(\hat{a},\hat{b},\hat{d}\right)$, given an acquired 2D digital image $I_{a}\left(i,j\right)$ of the lumen cross-section of known *R*, *w*, and $\Delta_{s}$. This image is the input to the CNN, which approximates the mapping from the image space to the parameter space. We will compare the performance of two approaches to parameter vector estimation: least-squares (LS) model fitting and prediction by a convolutional neural network [31]. Using the former method, the lumen contour parameters can be estimated by minimizing the sum of squared differences between the acquired and modeled image samples:

(13) $$\hat{p}=\arg\left(\min_{p}\sum_{i=-N}^{N}\sum_{j=-N}^{N}{\left[I_{a}\left(i,j\right)-I\left(i,j;p\right)\right]}^{2}\right).$$

The Scipy (version 1.11.4) Python module was used to perform minimization of the sum-of-squares in (13).

### 2.5. Quantitative Evaluation of Contours Similarity

The lumen contour is defined by a closed continuous curve on the cross-section plane in a physical space. For numerical analysis, it is represented by a finite set of discrete point—vertices of a polygon chosen to approximate the curve. We will use the term “contour” to refer to its discrete version. We applied two measures to evaluate the discrepancy between contours. Consider the contours *A* and *B*, comprised of |A| and |B| points, respectively, resampled to $A^{\prime }$ and $B^{\prime }$. The mean distance between them is defined as

(14) $$mDist\left(A,B\right)=\frac{1}{\left|A|+|B\right|}\left(\sum_{a\in A}\underset{a\in A}{\inf}d\left(a,B^{\prime }\right)+\sum_{b\in B}\underset{b\in B}{\inf}d\left(b,A^{\prime }\right)\right)$$

where d(x,Y) is a set of Euclidean distances between a given point *x* and each of the points in set *Y*. In our experiments, we interpolated the contours being compared such that their cardinality was |A| = |B| = 100 and $|A^{\prime }|$ = $|B^{\prime }|$ = 500.

The second measure was the Dice Similarity Coefficient (DSC), computed for the sets A and B. These sets denote all the points of a common contour plane that lie inside the contour *A* and *B*, respectively. The DSC is defined as

(15) $$DSC=\frac{2|A\cap B|}{\left|A|+|B\right|}.$$

In our numerical experiments, the sets A and B were approximated by all the points of a high-resolution image (e.g., 800 × 800 points, coplanar with the contours), which were within a polygon defined by the points *A* and *B*, respectively.

### 2.6. CNN-Based B-Spline Model Parameter Estimation

**Figure 6:**
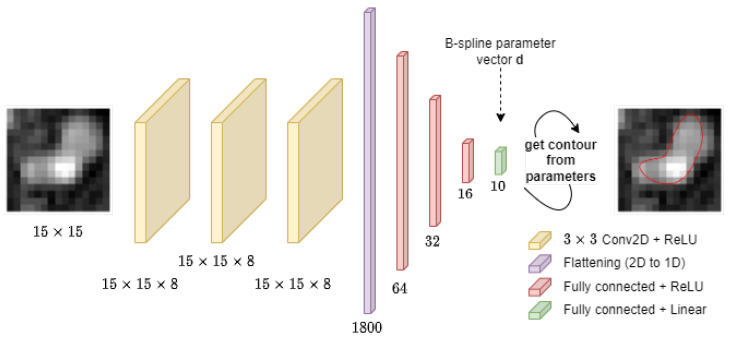
The neural network architecture used in the described experiments.

In the proposed method, the CNN plays a role of a nonlinear regressor. Its input is a lumen cross-section image. It predicts the values of the image model parameters at the output. A feedforward network was trained to predict the B-spline parameter vector **d**. The architecture is shown in Figure 6. Three 2D convolution layers are followed by a flattening layer and by four fully connected layers. The three convolutional layers feature 3 × 3 kernels, with a kernel number equal to 8 for each layer, “same” input padding type, and ReLU nonlinear activation function [52]. The flattening layer transforms the multichannel 2D feature maps into vectors. The vectors are then processed by three fully connected layers with 64, 32, and 16 neurons, respectively, and ReLU activation. The output layer estimates the 10 elements of vector **d** and features a linear activation function. In total, the network has 119,290 trainable parameters and was implemented in *keras* (version 2.13.1).

Network training was performed with the mean squared error as the cost function, optimized using the Adam algorithm [53]. Mini-batches of 64 examples were used during training. The maximum number of training epochs was set to 500, but an early stopping algorithm was employed to stop training before overfitting occurred. The early stopping patience parameter was set to 15 epochs.

The synthetic dataset was used to train the network for prediction on the PAVES dataset. For the test set, 10% of the data was excluded, which yielded 9000 examples. The validation set used in the early stopping algorithm was extracted from the training set as its 20% fraction.

The CAT08 dataset used for neural network training, validation, and testing has 652 examples. Of these, 66 (10%) were excluded for the test set, and the rest were split into training and validation sets in the same proportion of 90:10. Targets in the form of B-spline-approximated expert contour annotations were separated into three sets—one for each expert. The training procedure was repeated for each expert’s target set, yielding three trained models.

In summary, five models were trained: two for the PAVES datasets (noiseless and noisy) and three for the CAT08 datasets. These models are evaluated in the Results.

### 2.7. Methods and Tools for Statistical Analysis of the Results

**Figure 7:**
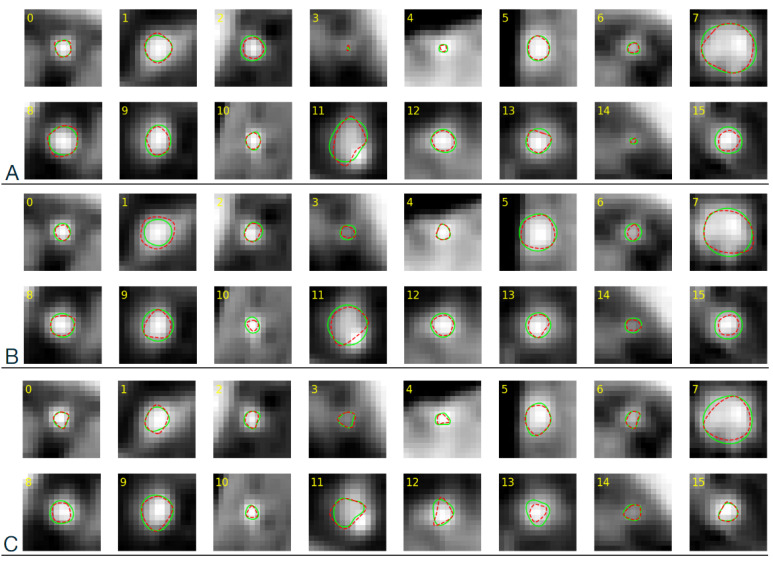
Example sixteen images from the CAT08 data test set. Red line: contours marked by an observer, green line: CNN-predicted contours. (**A**) Observer 1, (**B**) Observer 2, (**C**) Observer 3.

The cross-sections of coronary arteries visualized in the CAT08 dataset were annotated by three observers. Each of them delineated the lumen boundaries, presented as polygons on cross-section planes. The polygon vertices are available in the dataset. The three trained CNNs were applied to the same images to generate corresponding lumen contours. For each test image from the test set of $N_{T}=66$ images, two contours were then available, one marked by an observer and the other predicted by the corresponding CNN instance (Figure 7).

The image area covered by each contour was computed to give $3\times N_{T}$ = 198 pairs of values—$Area_{Obs}$ and $Area_{CNN}$, respectively, for the observer and for the CNN. These are the data points. Since they relate to the same image space, they are paired and match each other. In the context of this study, the question is whether the two methods of marking the lumen contour (by the observer and by the CNN) differ [54]. This question can be answered by a paired statistical test. The choice of a particular test has to be preceded by a test for the normality of the data populations. D’Agostino and Pearson’s normality test, available as *scipy.stats.normaltest*() function in the *Scipy* (version 1.11.4) library, was applied for this study. The area measurements failed the normality test. Based on this result, the paired samples Wilcoxon test was applied to the data, *scipy.stats.wilcoxon*(). It is a nonparametric test of the null hypothesis that two samples come from the same population against an alternative hypothesis, as described and discussed in the Results.

**Figure 8:**
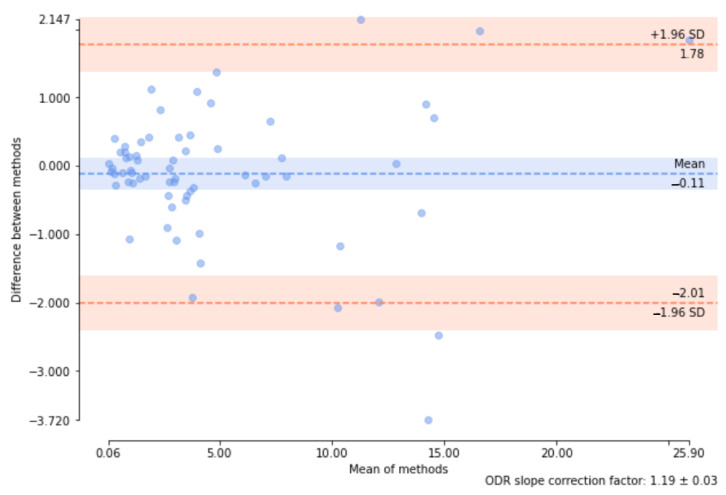
Bland–Altman plot for $Area_{Obs1}$ and $Area_{CNN1}$ over the test set. Computed with the ODR option of the *blandAltman*() function from the pyCompare library [56] to model and remove the multiplicative offset between each assay by orthogonal distance regression.

More quantitative information about statistical differences between the methods is available from the Bland–Altman plot which visualizes both bias and confidence intervals of the sample differences [55]. Figure 8 obtained with *pyCompare* library (version 1.5.4) is an example of such a plot.

All the computations in this work including statistical analyses were performed on a standard PC computer (Intel Core i5-8300 H, 2300 GHz, 16 GB RAM, NVIDIA GeForce 1050) under Windows 10 Home operating system in *miniconda*3 *Python* (version 3.10.13) programming environment. Occasionally, a laptop or an iPad Pro machines, of similar performance were used.

The above procedure was applied to the mDist (14) and DSC (15) coefficients which quantify the distances between contours marked by different methods, e.g., to test whether the populations of mDist data computed for contours marked by Observer 1 and Observer 2 and the mDist data for contours outlined by Observer 1 and Observer 3 are the same or different. Those tests are described and discussed in Results.

## 3. Results

### 3.1. PAVES Dataset

#### 3.1.1. LS Model Fitting to PAVES Data

Prior to delineating blood vessel contours in the PAVES images with the CNN-based technique, the performance of LS model fitting was evaluated. A twelve-parameter image formation model (12) was chosen. The adjustable parameters vector comprised (*a*, *b*, $d_{0},\ldots ,d_{9}$) entries, whereas the others were fixed at $\Delta_{s}$ = 1.0 mm, *R* = 11, $w/\Delta_{s}$ = 0.65. The solutions were searched in the range [0, 0.3] for *a*, [0.1, 0.5] for *b*, and [−0.3, 1.0] for each element of the vector d. A negative value of $d_{i}$ indicates that the corresponding control point $C_{i}$ is located on a radial line at the angle ($\phi_{i}+\pi$), cf. (10), (11).

**Figure 9:**
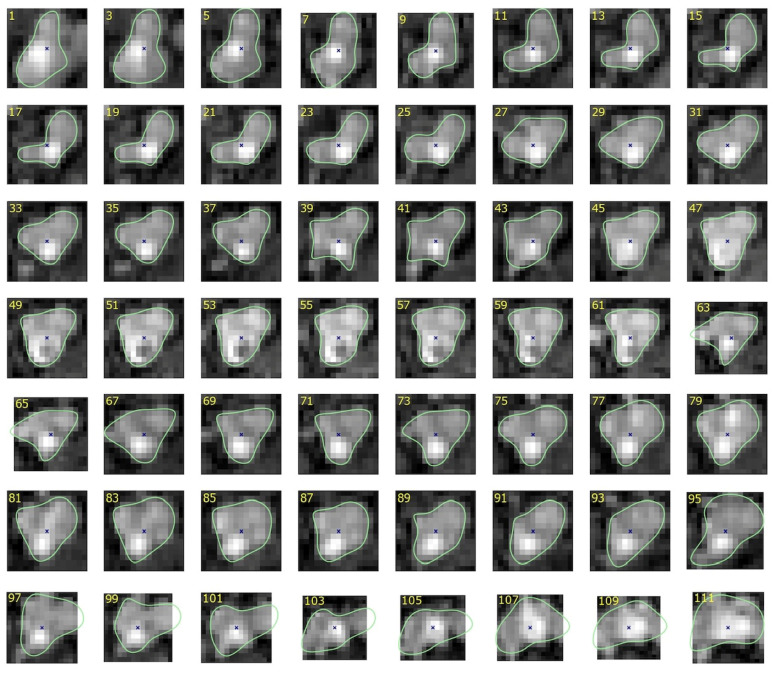
Example contours (light green lines) of the vein–artery lumen obtained via LS model fitting to 15×15-pixel odd-numbered (1, 3, …) sections of branch b14 in the PAVES 05 dataset. Twelve-parameter (*a*, *b*, $d_{0},\ldots ,d_{9}$) B-spline lumen model, $\Delta_{s}$ = 1.0mm, *R* = 11, $w/\Delta_{s}$ = 0.65. The points marked by a blue “x” symbol indicate the approximate centerline location.

We used the minimize() function from Scipy with the SLSQP method. This allowed the imposition of bounds on the estimated model parameter values. Estimation of the lumen model parameters for all 113 cross-sections of branch b14 (spaced at distances of 0.5 mm from each other) took about 3 h on an Intel Core i5 PC computer. The results are shown in Figure 9. As can be seen, there was a high level of consistency in the contour shape when moving between the consecutive cross-sections. Apparently, no significant local minima were encountered in this optimization experiment, although a robust starting point range for the elements of d was found after a few attempts, as [0.2, 0.4] for the collection of images.

The sections presented in Figure 9 feature a relatively flat background. In contrast, the intensity of the foreground representing both arteries and veins varies significantly within an image. Nevertheless, model fitting resulted in stable values of parameters *a* and *b*, with means (standard deviation) equal to 0.10 (0.009) and 0.18 (0.017), respectively.

**Figure 10:**
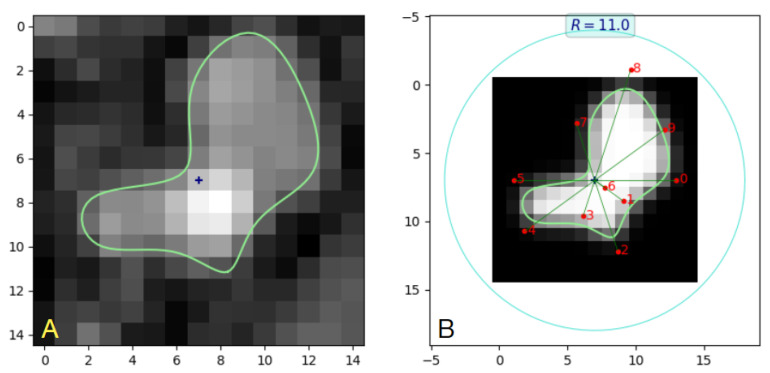
(**A**) B-spline contour example for Section 14 of branch b14 in PAVES 05. (**B**) B-spline geometry and synthesized image. The control points are marked by numbered red dots. Control point $C_{6}$ is located on the radial line at angle −36° (instead of 144°), which indicates a negative value of $d_{6}$. The LS-identified d vector for this image is (0.5, 0.23, 0.50, 0.25, 0.58, 0.54, −**0.08**, 0.40, 0.77, 0.57).

The d parameters spanned the range [−0.1, 1.0]. Negative values were found for parameter $d_{6}$ in sections 12–18 only, where the contour closely approaches the image center. An example illustration is shown in Figure 10 for section 14. The angle of the radial line for $d_{6}$ takes the value −36°, while it would be 144° for positive values. The fitting experiment was repeated a few times for different values of *R*. In the case of images where the lumen region was completely included in the image, with a reasonable margin in the background, e.g., sections 35–55, the values of d linearly scaled with 1/R. An increase in *R* causes a proportional decrease in all the elements of d. This property is not observed in areas where the lumen region goes beyond the image edge, such as in sections 97–112, e.g., in the neighborhood of the vessel bifurcations.

Scatter plots of pairs of vector d elements are presented in Figure S2. Only those involving $d_{6}$ occupy the range of values close to 0 or slightly negative. The plots demonstrate a clear correlation between the parameters. Some visual patterns can also be identified, suggesting the possibility of using these parameters as features for the classification of contour shapes.

Figure 2 shows lumen surface visualizations of branch b14 in PAVES data, LS-estimated with B-splines and circular-cross-sections image formation models. Clearly, the circularity assumption is not appropriate in the case of the PAVES data. It is, however, applicable and gives accurate lumen quantification in the case of artificially designed objects, such as physical phantoms for MR scanners calibration [31].

#### 3.1.2. Generation of Synthetic Images for CNN Training

To train the neural network to estimate lumen contour parameters for the PAVES data, we computed synthetic images. The 12-parameter model (12) was used for this purpose. The parameter values were randomly drawn from a uniform distribution over [0, 0.2] for *a* and [0.2, 0.3] for *b*. The 10 parameters in d were drawn from the range [0.0, 1.0] and treated as a circular sequence, smoothed with a simple lowpass filter [0.15, 0.7, 0.15]. This step introduces correlation into the d vector, to simulate their bonds to the contour shape. The value of one in ten parameters was randomly drawn from the range [−0.2, 0.1], with probability 0.25 (corresponding to the observed rate of negative d values in PAVES data). This takes account of concave contours. Details concerning their structure are presented in Figure S3.

#### 3.1.3. CNN-Estimated B-Spline Model for PAVES Data

**Figure 11:**
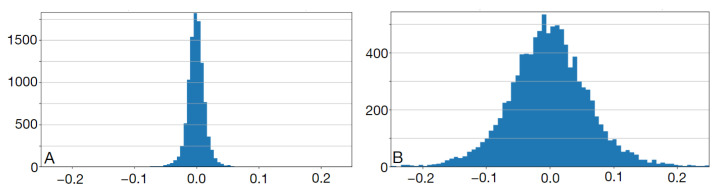
Histogram of the differences between CNN-estimated and true values of B-spline parameters over the test sets of 1000 images. Training sets size: 8100 images. (**A**) noiseless case, CNN trained for 154 epochs and (**B**) noisy case, CNN trained for 204 epochs.

The neural network was first trained on the synthesized noiseless images in the training set. Figure 11 shows a histogram of the error in B-spline parameters estimation over the test set. This error has a normal distribution and its mean is practically zero—the network does not introduce any bias. This applies to individual parameters as well. The standard deviation was approximately 0.02.

**Figure 12:**
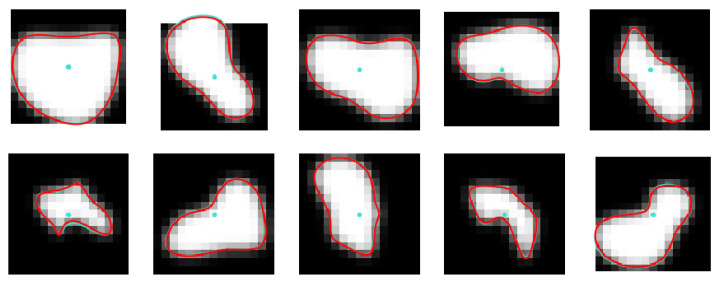
Examples of noiseless images of the test set, synthesized with the use of randomly generated B-spline parameters. Contours computed from ground-truth parameters and their CNN-predicted estimates are drawn using turquoise and red lines, respectively. Turquoise lines are practically invisible as they are closely matched by the red ones.

Figure 12 contains example images from the noiseless synthetic test set with two lines superimposed on them. The turquoise line is a randomly generated contour used for image computing with the use of expressions from (1)–(12), whereas the red curve represents the contour corresponding to B-spline parameters predicted by the CNN. The input to the CNN was the corresponding image in each case. As can be seen, the two lines overlap basically, indicating excellent accuracy of the CNN predictions. This was confirmed by the very small values of mDist and practically unit values of DSC measures applied to the two contours over the synthetic test set.

**Figure 13:**
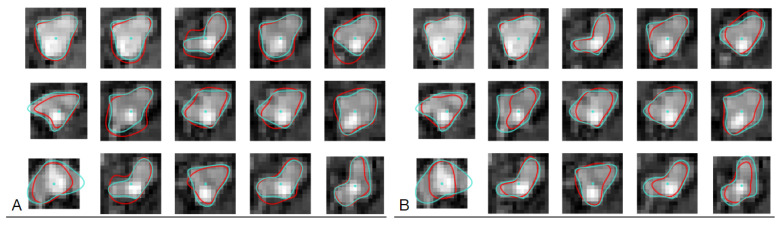
Example sections of PAVES b14 branch, taken at random. Turquoise lines: lumen contours obtained via LS model fitting. Red lines: contours computed using B-spline parameters predicted by CNN trained on synthesized noiseless images (**A**) and noisy images (**B**).

The CNN trained on the synthesized noiseless images was applied to the cross-sections of branch b14, visualized in PAVES MR volumes. For each cross-section, the network predicted a set of B-spline parameters which was used to compute coordinates of the estimated lumen contour points. Example results are shown in Figure 13A. There is generally good agreement between the contours obtained with the use of the proposed method and the LS model fitting. However, spurious bright objects present in the background, e.g., in the rightmost image in the upper row, “attract” the CNN contour, which as a result departs from the LS-identified curve. This deviation can be attributed to the CNN’s training on idealized images, with constant values for intensity inside the lumen region and in the background. During this training, the network did not acquire any information about possible spatial variations in image intensity. More realistic training examples are therefore needed.

**Figure 14:**
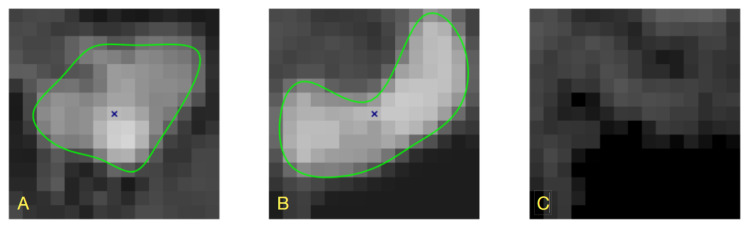
(**A**) Example Section 27 of PAVES b14 branch with LS-predicted lumen contour. One can note random intensity variations. (**C**) Sample of GIMP-generated fractal-like texture, visually similar to patterns observed in the PAVES data. (**B**) The image in (**C**) added to a noiseless image synthesized using the contour marked by the light green line.

To accommodate image intensity variations over the training dataset, we added patch samples of random fractal-like texture to the synthesized noiseless images. The texture patterns and intensity were chosen to visually resemble the properties of PAVES cross-sections (Figure 14). The created images were collected to constitute a synthesized noisy training set and test sets (10,000 examples in total).

The neural network was trained on synthesized fractal-noise images. Figure 11B shows a histogram of the error in the estimation of B-spline parameters over the synthesized noisy test set. Similarly to the noiseless case, the error has a normal distribution and its mean value is close to zero (cf. Figure 11). However, its standard deviation is a few times larger. At this expense, the contours are less dependent on the intensity variations and, visually, approximate the true boundaries better (see Figure 13B where the CNN trained on the noisy dataset was used to predict contour parameters of PAVES sections).

### 3.2. CNN-Based Modeling of Artery Lumen in CAT08 Dataset

Our first attempt to find the lumen contour of the blood vessels visualized in the CAT08 images involved LS-fitting of the image formation model by numerical minimization of the goal function (13). The parameter vector comprised twelve elements: *a* and *b* for background and lumen intensities, respectively, and d = ($d_{0}$, *…*, $d_{9}$) for the contour shape. Despite numerous trials tuning the meta-parameters of the optimization routine, no satisfactory result was obtained. After time-consuming computations, the optimized contours were located far from the observers’ markings. This approach to CAT08 non-circular lumen quantification was therefore abandoned. To train the CNN for contour shape quantification, we assumed the parameters *a* and *b* in the image formation model would not be estimated (although they could be if needed [31]). The CNN was then trained to ignore the natural variation in background and lumen intensities in the training cross-section images. The training examples comprised pairs of 2D cross-section images (with their middle points positioned at the geometrical centers of contours marked by the three observers on the common plane), and the B-spline parameters of the marked contours.

**Figure 15:**
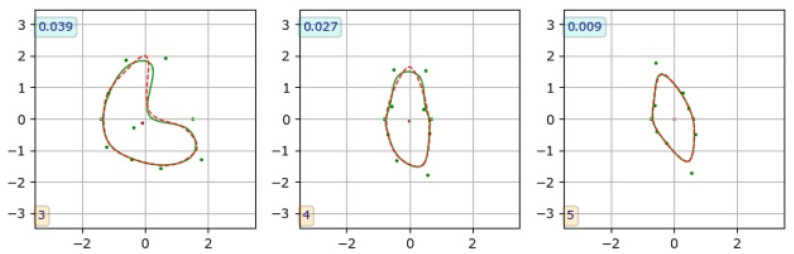
Three examples of fitting B-spline curves to observer-marked contours (CAT08 data), to find B-spline parameters for CNN training. Dashed red line: contour marked by an observer, green line: B-spline curve. The numbers in boxes indicate the corresponding mDist value.

A contour marked by an observer is available in the CAT08 dataset as a set of ($x_{i},y_{i}$) coordinates, i∈{0,…,Q} of discrete points, where *Q* is the coordinate count for different contours. To obtain the B-spline parameters for a contour, we used numerical minimization of the mean distance (14), where *A* is the observed contour and *B* is a B-spline curve. The course of the B-spline curve was controlled by the entries for the parameter vector d. This process took a fraction of a second per contour executed with a Carnetplus (version 1.8.0) Python library installed on an Apple iPad Pro tablet (11inch, 3rd generation, iPadOS 17.2), resulting typically in mDist≊0.01 at optimum values of d, Figure 15, right panel. Figure 15 shows two of the worst results (the left and middle plots). As can be seen, the contour similarity is still excellent for the task at hand.

Slightly better results in terms of lower mDist values were obtained with NURBS contours [49,50], mainly for highly curved lines such as those shown in Figure 15 on the left and in the middle. This was achieved with the use of an *geomdl* library (version 5.3.1) at the expense of an increased number of model parameters (the node weights). No significant fit accuracy was observed for most lumen contours extracted from low-resolution images, which were rather smooth. The use of NURBS was therefore deemed unnecessary for the data analyzed in this particular project.

The artery cross-section images from each observer were fed into the CNN as input. The B-spline parameters found for the observer-marked contour were used as the target in the training process described in the Materials and Methods section. Thus, three CNNs were trained on the CAT08 dataset. A typical histogram of the differences between CNN-estimated and observer-contour-related values (for Observer 1) is shown in Figure S4. As can be seen, the estimation error is similar to those described above for the CNN trained on synthesized images.

**Figure 16:**
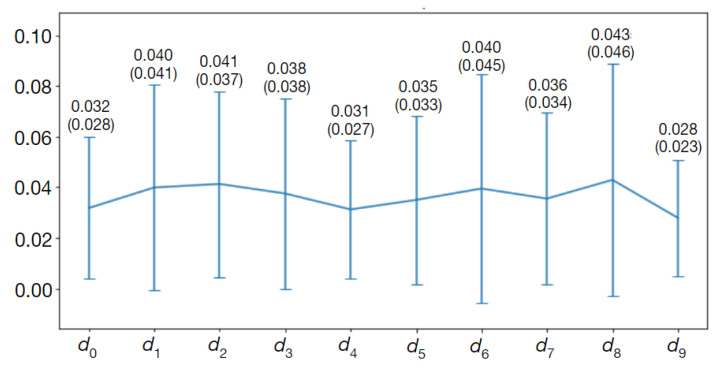
Plot of the mean values and standard deviations (numbers in brackets) of mean absolute differences between CNN-estimated and Observer 3 contour-related B-spline parameters over the test set of 66 images (CAT08 data). CNN trained on 527 images for 199 epochs.

Figure 16 shows a graphical representation of typical mean values and standard deviations of the mean absolute error (MAE) for the CNN-predicted d vector elements over the test set. The mean values of MAE oscillate around 0.035. The scale factor chosen for this experiment was R=10 and the sampling interval was $\Delta_{s}=0.45$ mm. The mean values of MAE over the test set are approximately equal to 0.16 mm and their standard deviations are less than 0.20 mm. The distance of B-spline control points from the common center point can thus be determined with subpixel accuracy.

**Figure 17:**
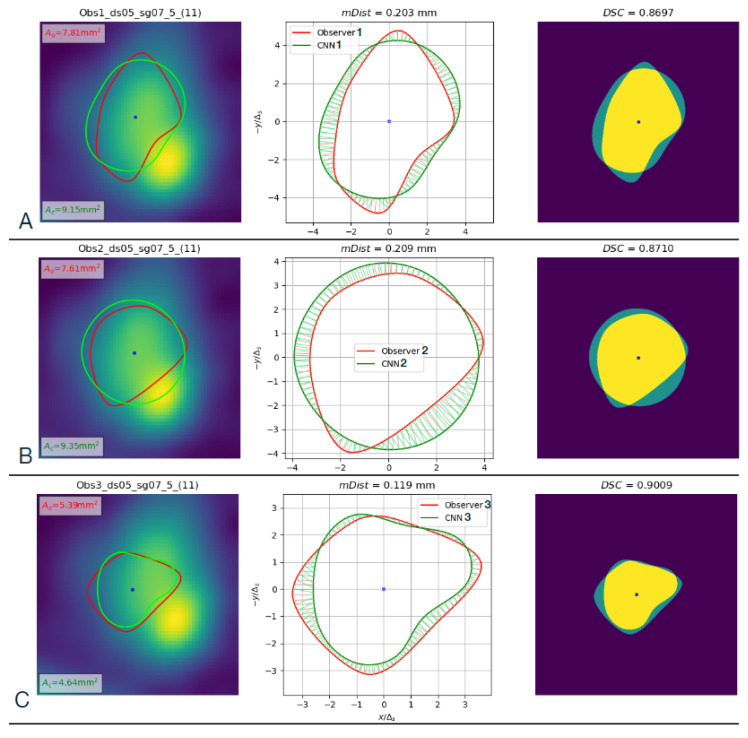
**Left column**: Comparison of contours marked by the three observers and computed with the corresponding CNN-predicted B-spline parameters for cross-section 11 in the test set (CAT08 dataset 05, segment 07, Section 5). (**A**) Observer 1, (**B**) Observer 2, (**C**) Observer 3. **Middle column**: Geometric illustration of mDist calculation. **Right column**: Image regions used to compute the DSC.

The contours marked by the observers and identified by the CNN are depicted in Figure 7, which shows examples of cross-sections sampled from the test set. Despite variations in the shape and size of the contours, there is consistent visual similarity between the curves marked by each observer and identified by the corresponding CNN. It may also be noted that the shape of the observer-marked contours on a given image differs in some cases for different observers, see, e.g., slices 11 and 12 (fourth and fifth from the left in the lower rows). Figure 17 illustrates the differences for section 11. The mDist (14), DSC (15), and image area inside the contours were computed to quantify similarities and differences (Table 1). These measures are complementary. mDist reflects the smallest absolute distances between contour points and does not depend on the number or size of the contours. DSC expresses overlap between areas enclosed by the contours, divided by the sum of the areas. As such, it can take large values for small-area lumen sections which are deformed or shifted only. The area is an absolute measure of contour size and does not reflect its shape.

As can be seen from Table 1, the CNN was in a better agreement with Observer 3 than with the other experts: mDist was almost two times smaller, DSC was larger by ca. 0.03, and the contour area differed by about 14%—compared to 20% and 23% for Observers 1 and 2, respectively. On the other hand, the average area for Observer 3 and its neural network model was only 60% of the average of those marked by the other two experts and the CNNs trained on their data. Interestingly, in agreement with the observers, all three CNNs learned to recognize a lower-intensity region close to a bright spot as the lumen area, see, e.g., cross-section 11 in Figure 7. This is not incidental, as there are other cross-sections with this feature in the test set which were correctly delineated by the CNN in this way.

#### Analysis of Experimental Results for CAT08 Dataset

**Figure 18:**
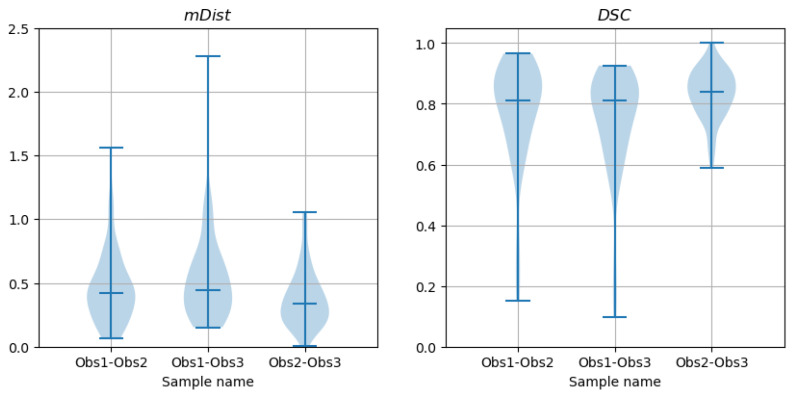
Violin plots of mDist and DSC descriptors of differences between contours marked by different observers on CAT08 images (interobserver differences).

The values of mDist and DSC coefficients were computed for all 66 images in the test set. Figure 18 shows the violin plots for each observer–observer pair. The statistical distribution is not normal, as was confirmed by D’Agostino and Pearson’s normality test applied to all six samples presented in the figure. The median values, listed in Table 2, are marked on the plots with horizontal lines.

To compare the mDist and DSC coefficients computed for contours marked by different observers on the same image sections (Figure 18), the paired samples Wilcoxon test was conducted. The results for all six combinations of samples rejected the $H_{0}$ hypothesis that the paired samples represent the same distribution. Most likely, each mDist sample comes from a different distribution and the distributions of DSC samples are also different from each other. These results indicate significant differences between the lumen contour shapes inferred by the observers from the cross-section images. The corresponding sample median and mean values are listed in Table 2.

**Figure 19:**
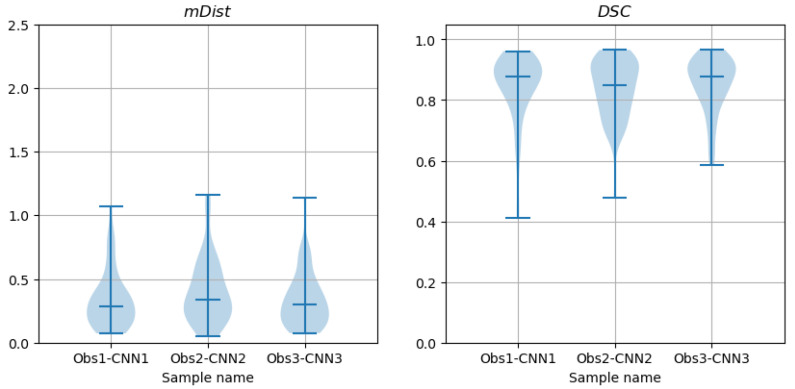
Violin plots of mDist and DSC descriptors of differences between contours marked by observers on CAT08 images and computed from CNN predictions (trained on the corresponding observer data) over the test set.

The paired samples Wilcoxon test applied to observer-CNN mDist (Figure 19) failed to reject the $H_{0}$ hypotheses that the samples Obs1-CNN1 paired with Obs3-CNN3 come from the same distribution, and that Obs2-CNN2 paired with Obs3-CNN3 come from the same distribution. However, it rejected the $H_{0}$ hypothesis (*p*-value = 0.013 with a significance level of α=0.05) for Obs1-CNN1 paired with Obs2-CNN2. The Wilcoxon test did not reject the $H_{0}$ hypothesis in the case of paired Obs-CNN samples of the Dice coefficient DSC. Then, only one of the six tests indicated a significant difference between the sample distributions of the discrepancies between the CNN-predicted and observer-delineated contours.

Given that the mDist median values (and mean values) were smaller for the observer–CNN (Table 3) estimates than for the observer–observer contour shape estimates (Table 2), one can conclude that the average distance between the CNN-predicted contours and the contours marked by the observers is statistically smaller than the distance between the contours marked by different observers on the same image. Similarly, the median (and mean) values in Table 3 for DSC are larger than those in Table 2. Thus, the contour shapes drawn by the observer were closer to the shape of the curves predicted by the corresponding CNN than to the shape of contours delineated by an other observer.

**Figure 20:**
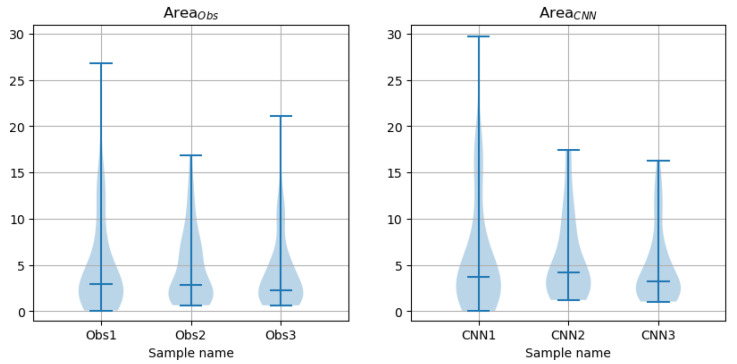
Violin plots for $Area_{Obs}$ and $Area_{CNN}$ in the test set.

Figure 20 shows violin plots for $Area_{Obs}$ and $Area_{CNN}$ contour descriptors. The sample median and mean values are listed in Table 4. Table 5 presents the results of related paired Wilcoxon tests. The *p*-values for inter-sample tests (first three rows in the table) show a very similar pattern. The $H_{0}$ hypothesis was rejected for Obs2 paired with Obs3 and the test failed to reject it for Obs1 paired with Obs2. This concerns both observer-marked contours and the CNN-produced values. The test for Obs1 paired with Obs3 failed with regard to observer contours and rejected the $H_{0}$ for the CNN contours. However, the *p*-values of the two tests (second row in Table 5) are close to the significance threshold α = 0.05.

Rows four to six in Table 5, containing *p*-values for paired tests of CNN and observer contours, indicate that the tests rejected the hypothesis that the samples of the coefficients $Area_{CNN}$ and $Area_{Obs}$ were drawn from the same distribution. Indeed, the Bland–Altman plots show that the mean values of the differences are substantial (see an example for $Area_{Obs1}$ paired with $Area_{CNN1}$ in Figure 8), even if detrending options are applied (Table 6).

The contour shape is encoded in B-spline parameters d (Section 2.3). As expected, the area inside the contour carries additional information about the contour size. This information should be explicitly included in the goal function of the CNN training. This step in the optimization of the CNN architecture and training strategy lies beyond the scope of this article and will be the subject of future research.

## 4. Discussion

This study has demonstrated that the integration of B-splines with advanced deep learning techniques significantly enhances the precision and reliability of blood vessel lumen modeling from 3D images. Based on two publicly available datasets, PAVES and CAT08, B-spline-approximated lumen contours were used in the image formation model to quantify the non-circular shape of blood vessels with subvoxel accuracy. Such accuracy cannot be obtained using existing circular or elliptical approximations.

The experimental results obtained for the PAVES dataset demonstrated good agreement between the B-spline parameters estimated by the CNN and those computed with the use of LS model fitting. It was shown that the differences can be reduced by proper augmentation of the synthesized images used for the CNN transfer training. Unfortunately, the PAVES dataset does not contain any reference information. No radiologists’ annotations are available, so there are no ground-truth contours to compare with those related to B-spline parameters found in the course of LS model fitting or CNN-based prediction. Still, the dataset was useful to demonstrate that the proposed lumen quantification approach, based on the image formation model, can be successfully applied to highly non-circular blood vessel cross-sections. Moreover, we demonstrated the possibility of both obtaining reasonable contours for real-life MR images with the use of a transfer-trained CNN and making the CNN predictions robust to spatial intensity variations by adding fractal texture patterns to the training images. Optimizing the augmentation strategy will be the topic of future research.

A ground-truth reference is available within the CAT08 dataset quantified with our method. Due to the complexity of CT images of coronary artery cross-sections, LS model fitting does not provide the expected stability. Even with the use of troublesome constrained optimization, it finds local minima too often and the fitting results depend heavily on the initialization points. In contrast, the CNN can be trained to ignore image nonidealities (e.g., bright objects in the background) and produce lumen contours which are consistent with the observers’ delineations. Of course, it is also much faster than human experts. Its application to image annotation would relieve the radiologist of this task.

Our experiments show that the CNN can be trained to mimic an individual observer’s style of contour marking. There was good agreement between the shapes of CNN- and observer-produced contours. The CNN reproduced the interobserver variability very well. This raises the question of the ground truth. Future work is planned to design and implement realistic digital and physical phantoms of known tubular geometry and blood distributions. These phantoms will be used to simulate and acquire images, which will then be evaluated by both radiologists and CNNs. The aim is to conduct a quantitative comparison of the respective contour delineations with the phantom properties.

There is a significant difference between the CNN and LS algorithms in terms of the computation time needed. When implemented on a moderate-performance PC (Intel Core i5 plus NVIDIA GeForce 1050), the CNN predicts the contour parameters in microseconds per image, compared to about 100 s for the iterative nonlinear LS program.

The CNN trained to predict the lumen model parameters has a simple architecture (Figure 6) and a relatively small number of adjustable weights. Its training on a dataset of 500 images requires only about 2 min on a standard laptop computer (MS Windows 11 Pro, Intel Core i5, 2400 GHz) with no GPU acceleration. Implementing the neural network is straightforward and does not present any major technical challenges. The proposed solution is much less computationally demanding than the majority of popular CNN applications.

Presumably, the efficiency of the proposed approach stems from the CNN being guided by the centerline while processing image samples. The CNN receives direct information about the distance of the sampling points to the lumen boundary. This information is clear-cut and encoded in the sampled intensity. Such a priori knowledge is built into our method. The CNN has only to approximate the inverse mapping from the intensity space to the point coordinates space, which is expected to be smooth (Appendix A).

## 5. Conclusions

Accurate geometric modeling of blood vessel lumen from 3D images is crucial for vessel quantification, as part of the diagnosis, treatment, and monitoring of cardiovascular diseases. It is a challenging task, given the complex geometry and internal structure of normal and pathological blood vessels, their widely varying diameters, and the limited capabilities of imaging sensors and scanners. The arteries and veins are composed of highly curved branches, interleaved in a space filled with various organs and tissues.

Manual vessel delineation by radiologists is time-consuming, tedious, and error-prone. The development of automated techniques for lumen quantification has been the subject of intensive research over the last few decades. Research has taken two main directions: 3D segmentation-based lumen quantification and model-fitting-based 2D lumen cross-section quantification along an approximate centerline. Binary segmentation introduces discretization errors, which may be critical for lumens with small diameters. Some segmentation algorithms require time-consuming iterative calculations. Therefore, we have followed the second approach.

Unlike other centerline-based methods, which assume a circular or elliptical vessel cross-section, the proposed method employs parametric B-splines combined with image formation system equations to accurately localize highly curved lumen boundaries. The parameters of this model are estimated using a feedforward convolutional neural network driven by full bit depth image samples on planes orthogonal to the centerline. This is in contrast to known B-spline-based lumen models which are identified from coarsely outlined binary regions in segmented images. The need for segmentation is avoided. Superior modeling accuracy of highly curved lumen boundaries in datasets comprising real-life MR and CT images is demonstrated.

The CNN, which has a rather straightforward architecture, can be trained on either computer-synthesized or expert-annotated images within just minutes using a standard desktop or laptop computer equipped with a standard graphics processor. The trained network predicts the parameter values in microseconds, compared to the minutes per image needed for alternative LS model fitting methods. High speed is thus one more advantage of the proposed estimator.

Due to its high speed, high accuracy and robustness, the proposed method can have an impact on and appear significant to medical diagnosis and research. It could be of great help to radiologists in their routine work. Also, the trained CNNs can be used for automated annotation of images in large datasets needed for the development of data-driven artificial intelligence solutions for healthcare. As the high accuracy of the vessel wall reconstruction is crucial for faithful blood flow simulation, this research area can benefit from using CNNs and B-splines for lumen modeling.

## Figures and Tables

**Table 1:** Descriptors of observer-marked and CNN-predicted contours in Figure 17. ^†^: Obs1 vs. CNN1, ^‡^: Obs2 vs. CNN2, *: Obs3 vs. CNN3.

	Obs 1	Obs 2	Obs3
mDist, mm	$0.203{\;}^{†}$	$0.209{\;}^{‡}$	$0.119{\;}^{*}$
DSC, −	$0.870{\;}^{†}$	$0.871{\;}^{‡}$	$0.901{\;}^{*}$
$Area_{Obs}$, mm^2^	7.81	7.61	5.39
$Area_{CNN}$, mm^2^	9.15	9.35	4.64

**Table 2:** Median/mean values of mDist and DSC descriptors of shape differences between contours marked by different observers on the same images in CAT08 over the test set.

	Obs1 vs. Obs2	Obs1 vs. Obs3	Obs2 vs. Obs3
mDist, mm	0.422/0.462	0.440/0.527	0.334/0.380
DSC, −	0.813/0.771	0.809/0.743	0.841/0.832

**Table 3:** Median/mean values of mDist and DSC descriptors of shape differences between observer-marked and CNN-predicted contours for CAT08 images over the test set.

	Obs1 vs. CNN1	Obs2 vs. CNN2	Obs3 vs. CNN3
mDist, mm	0.284/0.333	0.336/0.405	0.298/0.335
DSC, −	0.876/0.844	0.849/0.837	0.878/0.851

**Table 4:** Median/mean values for the contour area marked by observers $Area_{Obs}$ and predicted by CNNs $Area_{CNN}$ over the test set drawn from the CAT08 repository.

	Obs 1	Obs 2	Obs 3
$Area_{Obs}$, mm^2^	2.90/4.79	2.83/4.71	2.23/4.12
$Area_{CNN}$, mm^2^	3.73/5.83	4.18/5.68	3.26/4.82

**Table 5:** *p*-values of the paired Wilcoxon test for $Area_{Obs}$ and $Area_{CNN}$ coefficients over the test set drawn from the CAT08 repository.

	Observer	CNN	Observer vs. CNN
Obs1 vs. Obs2	0.775	0.751	−
Obs1 vs. Obs3	0.085	0.034	−
Obs2 vs. Obs3	3.5 × 10^−5^	1.9 × 10^−5^	−
Obs1 vs. CNN1	−	−	1.4 × 10^−8^
Obs2 vs. CNN2	−	−	1.2 × 10^−7^
Obs3 vs. CNN3	−	−	9.7 × 10^−9^

**Table 6:** Mean values (in mm^2^) of the differences between paired $Area_{Obs}$ and $Area_{CNN}$ coefficients over the test set drawn from the CAT08 repository. Columns ‘None’, ‘Linear’, and ‘ODR’ correspond to detrending options used in pyCompare() to compare the distributions [56].

	‘None’	‘Linear’	‘ODR’
Obs1 vs. CNN1	−1.04	−0.20	−0.11
Obs2 vs. CNN2	−0.98	−0.76	−0.33
Obs3 vs. CNN3	−0.70	−0.93	−0.65

**Figure A1:**
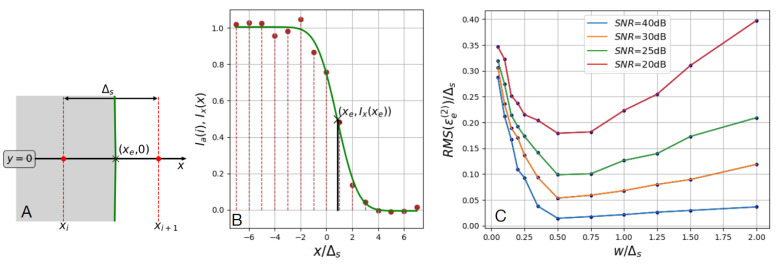
(A) Lumen boundary crossing the 0x axis between sampling points $x_{i}$ and $x_{i+1}$, i∈{−N,…,0,…,N−1}, noiseless case. (B) Computer-simulated image intensity profile along the 0x axis. Dashed red lines ended with dots: noisy image samples computed with the use of (A6) for a=0, b=1, $x_{e}=0.8\Delta_{s}$, $w=1.2\Delta_{s}$ and $\sigma_{\nu }\approx 0.032$ (SNR=30 dB). Continuous green line: image profile (A5) LS-fitted to the noisy samples, computed for optimum parameter values, a=−0.01, b=1.01, $x_{e}=0.84\Delta_{s}$ and $w=1.27\Delta_{s}$. (C) Root-mean-square values of the model-based edge estimation error $\epsilon_{e}^{\left(2\right)}$ computed 500 times for each of the 48 combinations of $w/\Delta_{s}$ and SNR.

## Data Availability

Data available in publicly accessible repositories. PAVES: https://paves.grand-challenge.org/ (accessed on 1 February 2019). CAT08: http://www.coronary.bigr.nl/stenoses/ (accessed on 14 February 2020).

## Supplementary Materials

The following supporting information can be downloaded at: https://www.mdpi.com/article/10.3390/s24030846/s1, Figure S1: S1_PAVES_coronal.png; Figure S2: S2_PAVES_d-scatter.png; Figure S3: S3_images_for_transfer_training.png; Figure S4: S4_cnn_error_ histo.png.

## Abbreviations

The following abbreviations are used in this manuscript:

CE	Contrast Enhanced
CNN	Convolutional Neural Network
CTA	Computed Tomography Angiography
DSC	Dice Similarity Coefficient
HU	Hounsfield units
LS	Least Squares
MAE	Mean Absolute Error
MRA	Magnetic Resonance Angiography
NURBS	Non-Uniform Rational B-splines
PSF	Point Spread Function
RMS	Root-Mean-Square
SNR	Signal to Noise Ratio

## Appendix A

This appendix presents the rationale behind the choice of model-based as opposed to segmentation-based approach to lumen geometry quantification, cf. Introduction. The main sources of errors in estimating the position of lumen edges from digital images are characterized. For clarity of presentation, the 2D image formation model defined in Materials and methods is reduced here to one-dimensional signal—the parameterized image intensity profile along a radial line 0x.

### Appendix A.1. Errors of Lumen Boundary Estimation from Segmented Image

Consider a small fragment of a cross-section image (Figure A1A) where the lumen boundary crosses the 0x axis at a point ($x_{e},0$). This point is located somewhere between two sampling points $x_{i}$ and $x_{i+1}$, $x_{i+1}>x_{i}$. The local radius of the lumen contour is assumed much larger than the sampling interval $\Delta_{s}$ and than the width of the imaging sensor impulse response domain. The lumen boundary is thus represented here by a straight vertical line. The image intensity inside the lumen [the shaded area in Figure A1A] is equal 1, while the background intensity (the white part) is 0. Negligible edge blur and noiseless case are assumed in Figure A1A, for clarity.

Let us examine the image segmentation through thresholding. The point $x_{i}$ lying in the high-intensity region is classified as belonging to the lumen, while $x_{i+1}$ is assigned to the background. The actual location of the boundary point $x_{e}$ is unknown − it is placed somewhere between the two (Figure A1A). Now presume the probability of any of its particular location between $x_{i}$ and $x_{i+1}$ is the same. If so, it is reasonable to take the average of *x* over this interval as an estimate of $x_{e}$

(A1) $$x_{e}^{\left(1\right)}=\frac{1}{\Delta_{s}}\int_{x_{i}}^{x_{i+1}}xdx=x_{i}+\frac{\Delta_{s}}{2}.$$

The error of $x_{e}^{\left(1\right)}$ is

(A2) $$\epsilon_{e}^{\left(1\right)}\left(x\right)=x-x_{e}^{\left(1\right)}=x-x_{i}-\frac{\Delta_{s}}{2},\;x_{i}\leq x\leq x_{i+1},$$

its maximum absolute value is $\Delta_{s}/2$, the average (expected value) is zero, the mean absolute

(A3) $$\epsilon_{MAE}^{\left(1\right)}=\frac{1}{\Delta_{s}}\int_{x_{i}}^{x_{i+1}}\left|x-x_{e}^{\left(1\right)}\right|dx=\frac{\Delta_{s}}{4}$$

and its standard deviation

(A4) $$\sigma_{e}^{\left(1\right)}=\sqrt{\frac{1}{\Delta_{s}}\int_{x_{i}}^{x_{i+1}}{\left(x-x_{e}^{\left(1\right)}\right)}^{2}dx}=\frac{\Delta_{s}}{\sqrt{12}}\approx 0.29\Delta_{s}.$$

It follows from (A4) that the only way to increase the precision of the estimator $x_{e}^{\left(1\right)}$ is to decrease the sampling interval $\Delta_{s}$. However, as pointed out in Introduction, this would deteriorate the image quality in terms of smaller SNR. There are technical and medical limits of the quality restoration, so there is a minimum practical value of the sampling interval for imaging devices and, consequently, a minimum attainable standard deviation $\sigma_{e}^{\left(1\right)}$. Methods of edge estimation with better precision than resulting from (A4) are of great importance and interest.

### Appendix A.2. Errors of Lumen Boundary Estimation by Model Fitting

Taking account of the assumptions of 2D Gaussian impulse response (5) and of large local radius of the lumen cross-section, one can show that the one-dimensional noiseless image profile $I_{x}\left(i\right)$ in the vicinity od the lumen edge can be approximated with the use of the well-known Gaussian cumulative distribution function φ(x)≡ erf (x), as follows

(A5) $$I_{x}\left(x\right)=a+\frac{b}{2}\left[1-\varphi \left(\frac{x-x_{e}}{w\sqrt{2}}\right)\right].$$

The noisy image samples acquired along the 0x axis can be modeled as

(A6) $$I_{a}\left(i\right)=a+\frac{b}{2}\left[1-\varphi \left(\frac{i\Delta_{s}-x_{e}}{w\sqrt{2}}\right)\right]+\nu \left(i\right),$$

where ν(i) represents the zero-mean Gaussian random noise of standard deviation $\sigma_{\nu }$.

Let **q** be the parameter vector in (A5), **q** = $\left(a,b,x_{e},w\right)$. Given the acquired noisy image samples $I_{a}\left(i\right)$, one can estimate its elements by minimising the sum of squared differences between the model $I_{x}\left(i\Delta_{s};q\right)$ and the corresponding samples

(A7) $$\hat{q}=\arg\left(\min_{q}\sum_{j=-N}^{N}{\left[I_{a}\left(i\right)-I\left(i\Delta_{s};q\right)\right]}^{2}\right).$$

where $\hat{q}=\left(\hat{a},\hat{b},{\hat{x}}_{e},\hat{w}\right)$. We define the edge location estimator for the model-based approach by

(A8) $$x_{e}^{\left(2\right)}={\hat{x}}_{e},$$

the third element of the optimum vector $\hat{q}$ in (A7).

Figure A1B shows an example of simulated noiseles image profile model (A5) fitted to the noisy image samples (A6). The LS-estimated edge position is $x_{e}^{\left(2\right)}$ = 0.84$\Delta_{s}$, close to the true value $x_{e}$ = 0.80$\Delta_{s}$. The error of the model-fitting estimator

(A9) $$\epsilon_{e}^{\left(2\right)}\left(x\right)=x-x_{e}^{\left(2\right)}$$

is $0.04\Delta_{s}$ only, whereas the error of the segmentation-based estimator $x_{e}^{\left(1\right)}$ would be equal to $\left(0.5\Delta_{s}-0.8\Delta_{s}\right)=-0.3\Delta_{s}$ in this example. This illustrates the model fitting advantage − for a given sampling interval the modulus of edge estimation error $\epsilon_{e}^{\left(2\right)}$ can be much smaller than in the case of $\epsilon_{e}^{\left(1\right)}$ for the segmentation-based estimation.

The subpixel-accuracy property of $x_{e}^{\left(2\right)}$ can be explained noting that the edge blur caused by the impulse response function that fulfills certain conditions [as (5) does], namely rotational symmetry of h(x,y) and positive-definite monotonic integral $I_{0}\left(x\right)=\int_{0}^{\infty }h\left(x,y\right)ydy)$, [57], unambiguously converts different distances between image points and the lumen edge to the corresponding image intensity values in [*a*,*b*]. Therefore, more information about the edge position, spread over the image samples, is available and the edge position can be estimated more accurately.

To further analyse the $x_{e}^{\left(2\right)}$ estimator properties, the edge fitting experiment was repeated for different values of $w/\Delta_{s}$∈ [0.05, 0.1, 0.15, 0.2, 0.25, 0.35, 0.5, 0.75, 1.0, 1.25, 1.5, 2.0] and a few values of Gaussian noise, SNR∈ [20, 25, 30, 40] dB. Given one of the 48 pairs $w/\Delta_{s}$ and the SNR, the true edge position was taken at random from [$0,\Delta_{s}$] with uniform probability distribution, and the model was fitted to the noisy samples to produce the corresponding estimated value of $x_{e}^{\left(2\right)}$. The process of drawing $x_{e}$, adding the noise and fitting the model was repeated 48 × 500 times.

The average of $\epsilon_{e}^{\left(2\right)}$ over all 24,000 numerical experiments was 0.003$\Delta_{s}$, with a maximum value of 0.027$\Delta_{s}$. Thus, the estimator can be considered practically unbiased. The $\epsilon_{e}^{\left(2\right)}$ root-mean-square (RMS) values of the error were computed and presented in Figure A1C. This error depends on the noise level. Consider a constant $w/\Delta_{s}$, e.g., equal to 1. The RMS error decreases from 0.23$\Delta_{s}$ to to 0.03$\Delta_{s}$, for SNR increasing from 20 dB to 40 dB. Taking account of (A4), one concludes the estimator $x_{e}^{\left(2\right)}$ is much more precise than the $x_{e}^{\left(1\right)}$, even for the substantial noise level of 20 dB. For noisy images, the precision of the latter would be even worse than characterized by (A4).

For a given noise level, the RMS error of $\epsilon_{e}^{\left(2\right)}$ attains a minimum in $w/\Delta_{s}$, around w∈ [0.5, 0.7]$\Delta_{s}$. This region corresponds to the largest absolute value of the derivative dφ(x)/d(x) in (A5) and (A6). For such a high-slope profile, any noise-induced average shift of the sampled intensity requires a little move of φ(x) along 0x to compensate for the mean intensity change. On the other hand, for larger values of *w*, the slope is low and larger horizontal displacement of φ(x) is necessary. Larger displacement increases the $\epsilon_{e}^{\left(2\right)}$ absolute value. For *w* tending to zero, the edge profile has even higher slope, however, the number of available samples with encoded distance to the edge, inside the narrower transition region, is small. This makes the estimator similar to $x_{e}^{\left(1\right)}$, featuring RMS errors close to (A4). These are interesting findings of practical value − the parameter *w* of an imaging sensor (the width of the impulse response domain) should be neither too small nor too large, if possible.
