# Supplementary material for: Using Deep Learning and B-Splines to Model Blood Vessel Lumen from 3D Images
Source: Sensors (Basel). 2024 Jan 28;24(3):846. doi: 10.3390/s24030846 (PMC10857344; doi:10.3390/s24030846)
Supplement: Supplementary file 1 [file sensors-24-00846-s001.zip › Sensors_supplement.pdf]

**Supplementary materials** to "Using Deep Learning and B-Splines to Model Blood Vessel Lumen from 3D Images" by Andrzej Materka and Jakub Jurek

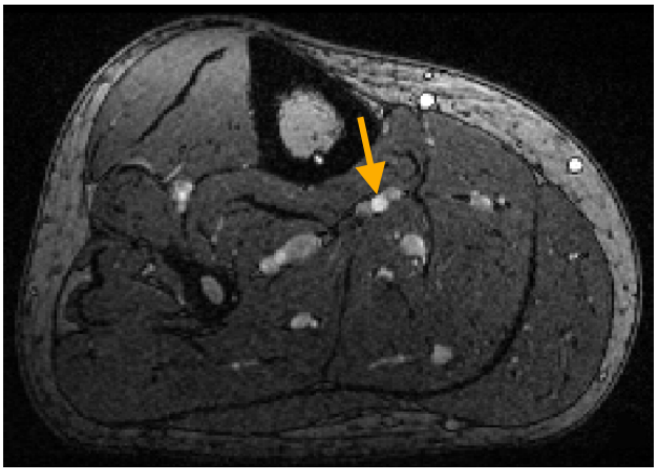

Figure S1. Coronal slice of a gadolinium contrast-enhanced (GdCE) MR 3D image (PAVES, dataset no. 2) of the lower part of a volunteer right leg. The arrow marks a pair of veins (gray ovals) on the left and right side of an artery (brighter circular blob). Fileneme: S1_PAVES_coronal.png file

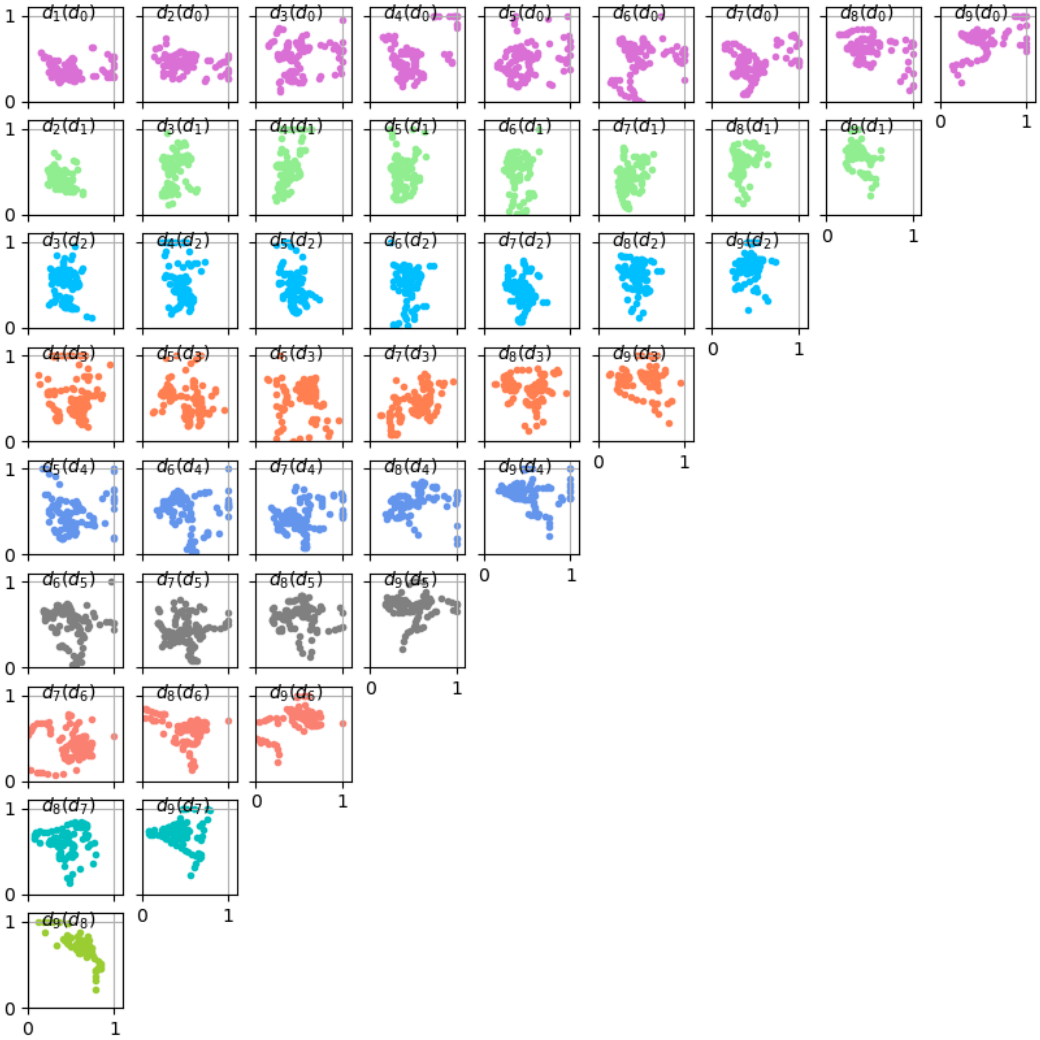


Figure S2. Scatter plots for LS-estimated B-spline contour parameters **d** for all branch b14 cross-sections in PAVES 05 dataset. Each subplot is annotated with a "$d_i(d_k)$" text meaning $d_k$ and $d_i$ are its horizontal and vertical coordinates, respectively. Fileneme: S2_PAVES_d-scatter.png

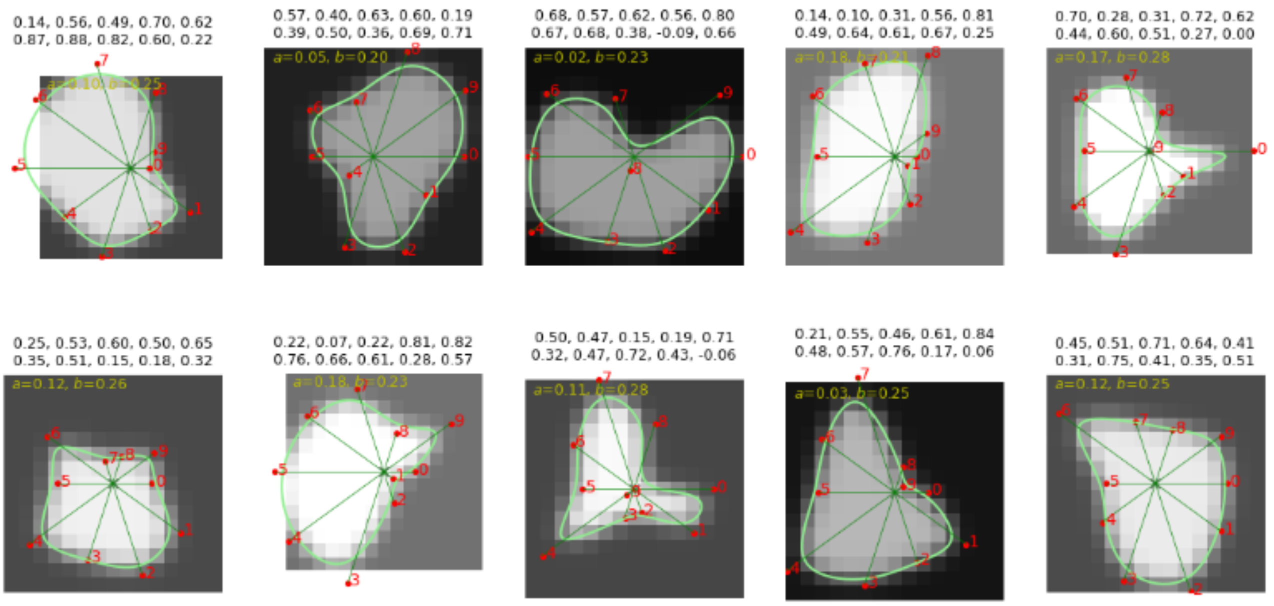


Figure S3. Examples of computer-simulated noiseless images for CNN transfer training. Green lines ended with numbered red dots represent $D$ = 10 elements of B-splines parameter vector **d** = ($d_0$, $d_1$, ..., $d_9$), cf. Figure 4A. Red-color digits plotted next to the dots denote corresponding indices of the parameters. The numbers above each picture are the values of these elements which were used to compute the corresponding image. Fileneme: S3_images_for_transfer_training.png

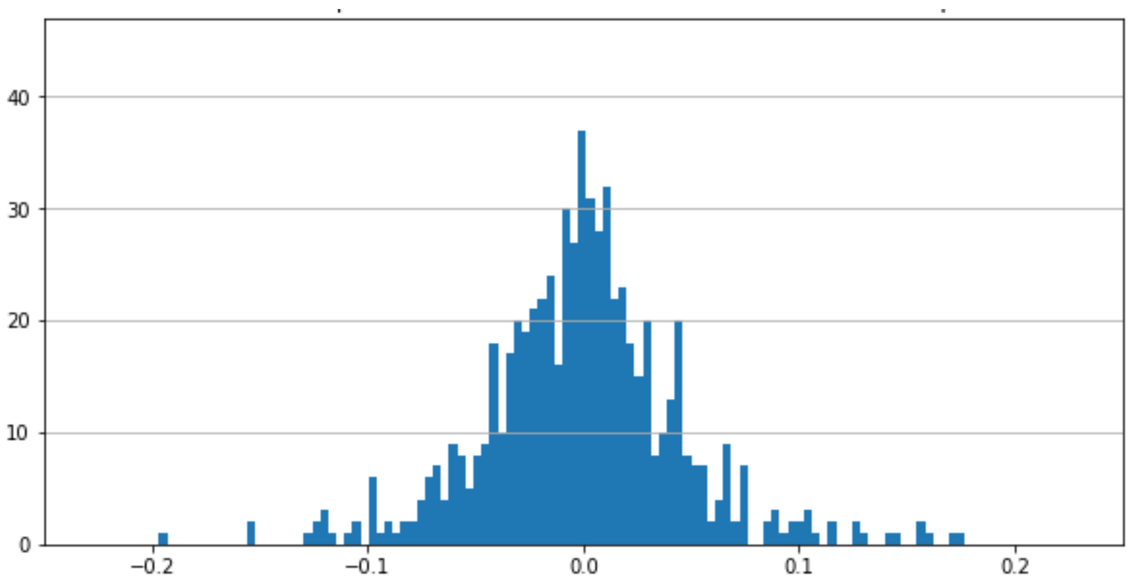


Figure S4. Histogram of the differences between CNN-estimated and Observer 1-contour-related values of B-spline parameters **d** over the test set of 66 images (CAT08 data). Pooled results obtained for all $D$=10 elements of the parameter vector **d**. The network was trained on 527 images for 184 epochs. Fileneme: S4_cnn_error_histo.png
