# Supplementary figures and images for: Using Deep Learning and B-Splines to Model Blood Vessel Lumen from 3D Images
Source: Sensors (Basel). 2024 Jan 28;24(3):846. doi: 10.3390/s24030846 (PMC10857344; doi:10.3390/s24030846)

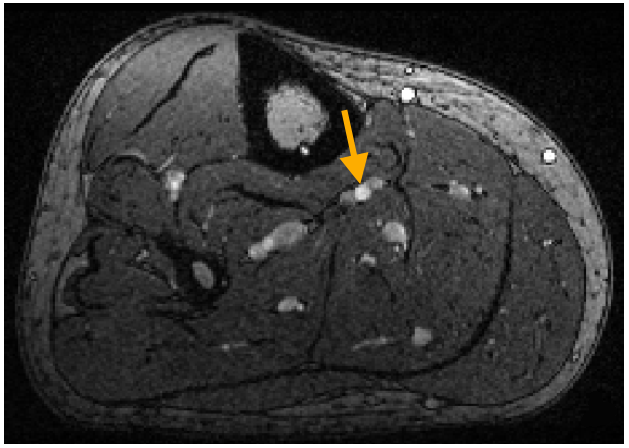

Supplement: Supplementary file 1 [file sensors-24-00846-s001.zip › S1_PAVES_coronal.png]

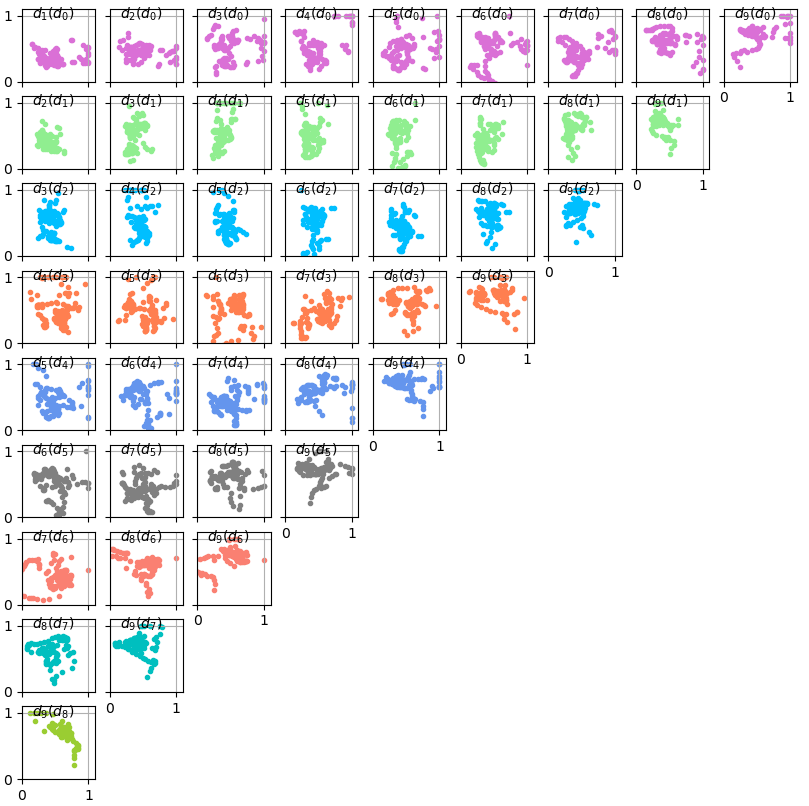

Supplement: Supplementary file 1 [file sensors-24-00846-s001.zip › S2_PAVES_d-scatter.png]

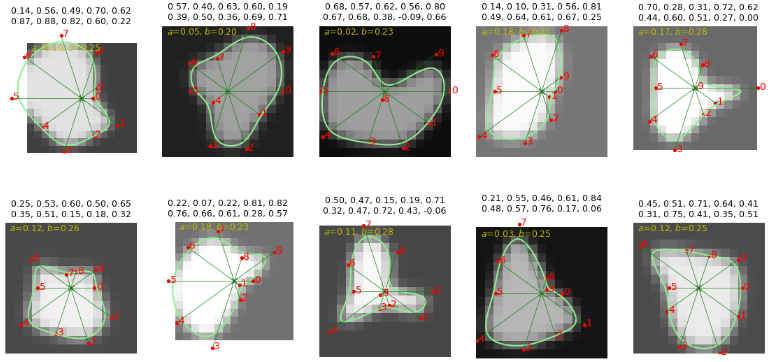

Supplement: Supplementary file 1 [file sensors-24-00846-s001.zip › S3_images_for_transfer_training.png]

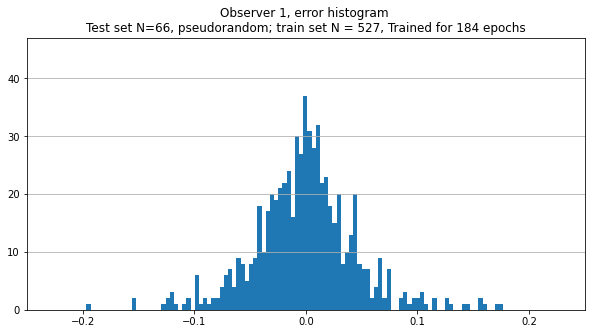

Supplement: Supplementary file 1 [file sensors-24-00846-s001.zip › S4_cnn_error_histo.png]
